# Mapping the Dynamics of Generalized Anxiety Symptoms and Actionable Transdiagnostic Mechanisms: A Panel Study

**DOI:** 10.1155/da/1885004

**Published:** 2025-05-13

**Authors:** Asle Hoffart, Nora Skjerdingstad, René Freichel, Sverre Urnes Johnson, Sacha Epskamp, Omid V. Ebrahimi

**Affiliations:** ^1^Department of Psychology, University of Oslo, Oslo, Norway; ^2^Research Institute, Modum Bad Psychiatric Center, Vikersund, Norway; ^3^Department of Experimental Psychology, University of Oxford, Oxford, UK; ^4^Department of Psychology, University of Amsterdam, Amsterdam, Netherlands; ^5^Center for Urban Mental Health, University of Amsterdam, Amsterdam, Netherlands; ^6^National University of Singapore, Singapore

**Keywords:** cognitive-attentional syndrome, generalized anxiety disorder symptoms, network analysis, panel data

## Abstract

**Background:** The long-term dynamic interaction between symptoms of generalized anxiety disorder (GAD) and their theorized mechanistic processes derived from three treatment models of GAD—the emotion dysregulation model, the model underlying metacognitive therapy (MCT), and the intolerance of uncertainty model—was investigated.

**Methods:** Four data waves 2 months apart were delivered by a representative population sample of 4361 participants during the COVID-19 pandemic in Norway. Networks were estimated using the newly developed panel graphical vector autoregression (panel-GVAR) methods.

**Results:** In the temporal network, and consistent with processes stipulated in the metacognitive model, the experience that worry is uncontrollable predicted the GAD symptom fear of awful events, which in turn predicted a range of other GAD symptoms, that is, anxiety, restlessness, and irritability. Fear of awful events had high outstrength, that is, predicted other variables to a large degree. Inconsistent with the metacognitive model, the coping strategy thought suppression negatively predicted restlessness. Consistent with the emotion dysregulation model, emotion dysregulation predicted avoidance. No relationships proposed by the intolerance of uncertainty model of GAD were identified in the temporal network. The contemporaneous network was dense with nodes clustering according to the constructs they belonged to.

**Conclusions:** The findings indicate the importance of the theory-derived variables, the experience and belief that worry is uncontrollable and emotion dysregulation, as potential targets for intervention to alleviate GAD symptoms. The findings also indicate that uncontrollability of worry and fear of awful events should be considered central symptoms of GAD in a within-individual diagnostics supplementary to current diagnostic manuals, such as the Diagnostic and Statistical Manual of Mental Disorders 5th edition (DSM-5).

## 1. Introduction

Generalized anxiety disorder (GAD) is distressing, impairing, and common, showing a lifetime prevalence of 3.7% [1, 2]. The Diagnostic and Statistical Manual of Mental Disorders, 5th edition (DSM-5 [1]) criteria include the core symptoms excessive anxiety and worry, difficulties controlling the worry, and associated symptoms related to mental and bodily functioning (e.g., trouble relaxing, restlessness, and irritability). The International Classification of Diseases, 11th revision (ICD-11 [3]) criteria are similar, except that uncontrollability of worry and being easily fatigued are not mentioned and subjective experience of nervousness and sympathetic autonomic overactivity are explicitly stated. Avoidance may also be a core dimension of GAD as avoidance has been found to be the most prevalent latent factor over time among persons with GAD [4].

There are several competing treatment models of GAD that emphasize different maladaptive mechanisms. For instance, GAD has been proposed to be the result of emotion regulation difficulties [5]. Individuals with GAD may experience high emotional sensitivity, difficulty identifying and understanding emotional experience, and have limited coping strategies to modulate their emotions. Therefore, their emotions are subjectively aversive, with a strong need to control them, leading to overreliance on control through worry, attempts to obtain security from others, and avoidance of situations with a possible negative outcome.

In the model underlying metacognitive therapy (MCT), GAD is linked to the activation of a particular maladaptive style of thinking referred to as the cognitive attentional syndrome (CAS [6]). The CAS is assumed to be a common pathway to several psychological disorders, with specific features related to specific disorders, such as GAD. It consists of cognitive perseveration, a thinking style that includes worry or rumination, attentional focus on threat, and unhelpful coping behaviors that have counterproductive consequences (e.g., thought suppression, situational avoidance). Worrying is directed to potential danger in the future (e.g., thoughts about being subject to rejection) and leads to exaggerated appraisals of threat and increased anxiety. More generalized worry (i.e., worry about many things) leads to wider threat monitoring, which biases information processing and thus inflates the sense of danger and anxiety. Threat monitoring relates to the detection of more dangers, thus extending the themes of generalized worry. Avoidance of perceived threatening situations is aimed at preventing threat and anxious thoughts and feelings but maintains anxiety by precluding corrective experiences of the dangerousness of the perceived threatful situation. Positive metacognitive beliefs drive the engagement in repetitive negative thinking and maladaptive behaviors through attributions of utility to the harmful process (e.g., “Worrying help me cope”). However, the repeated practice of worry contributes to a sense of loss of control over worry and to the development of negative metacognitive beliefs about the uncontrollability of worry (also a symptom of DSM-5 GAD [1]) and about the dangerousness of thoughts—for instance, the belief that some thoughts could lead to a loss of mind. These beliefs reinforce anxiety, and conversely, aspects of the anxious experience, such as racing thoughts, may be taken as evidence of loss of control and thus strengthen the beliefs.

As an alternative to the MCT model, the intolerance of uncertainty model of GAD posits that uncertainty is experienced as a threat by itself and is the cause of key GAD symptoms [7]. Worry is viewed as a strategy to remove uncertainty by mentally preparing for any eventuality; however, as complete certainty often is unachievable, worry persists and is easily experienced as out of control.

Thus, given the variety of proposed mechanisms, it is important to include them in one and the same study. Of further note, mechanistic relationships concern covariance between psychopathological processes and symptoms as they occur *within* individuals over time [8]. Investigating such dynamic within-person effects necessitates a collection of longitudinal data, in which potential psychopathological mechanisms and symptoms are repeatedly measured over extended periods.

All three models specify how observable processes and symptoms in GAD relate to each other (e.g., how worry leads to avoidance). Thus, they can be examined by applying a network analytic approach, which focuses on the interaction of components of phenomena [9–11]. Network analysis is a statistical tool enabling an examination of the unique association among multiple variables while controlling for all other variables in the system, further allowing an investigation of their predictive (i.e., temporal) relationships over time, as well as proximal (i.e., contemporaneous) within-person relationships. A few studies have examined temporal networks of predictive relationships between anxiety symptoms and proposed mechanistic processes on a daily or hourly time scale [12–14]. The evidence for the influential role of generalized worry has been mixed across these studies while intolerance of uncertainty showed an influential role in one study [14].

However, the relationship between anxiety components may differ across time scales. While daily experiences of anxious symptomatology provide important insights into the daily dynamics of the anxious condition, the long-term experience of such symptoms is one of the main prerequisites for a diagnosis [1]. Therefore, there is a need for studies on longer time scales, particularly because psychopathological processes and symptoms surpass over longer periods of time [1]. For instance, the A criterion of the DSM-5 diagnosis for GAD describes the presence of anxiety and worry “occurring more days than not for at least 6 months” [1].

Diagnostics of psychopathology (e.g., DSM) is traditionally based on between-person relationships. However, GAD symptoms are supposed to influence each other (e.g., worrying elicits anxiety) and the causal importance of symptoms can be settled only by studying the within-person dynamics [8]. Thus, a supplementary diagnostic based on within-person relationships between symptoms should be developed. Such a diagnostic is more clinically interpretable and has more direct implications for intervention [4].

However, within-person network dynamics on a monthly or yearly level have been difficult to study due to the lack of suitable analytic methods [15]. Recently, a new method for estimating networks from panel data, namely the panel graphical vector autoregression (panel-GVAR [15]), was introduced to fill this analytic gap in the literature, allowing the mapping of dynamic relationships between psychological phenomena on larger time scales.

The present study aims to use a panel network analytic approach to investigate the dynamic interaction between symptoms of GAD and their theorized mechanistic processes derived from the three theoretical perspectives presented above in an adult population sample across a 9-month period. Specifically, the study aims to investigate which of the theorized links between psychological mechanisms and symptoms from these three competing frameworks are supported by the data and to examine the interaction between GAD symptoms.

## 2. Methods

The study is part of the Mental Health and Adherence Project (MAP-19), a longitudinal survey of the general Norwegian population throughout the COVID-19 pandemic [16]. Measures and item selections along with the rationale and analytical plan of the study were preregistered before the data were analyzed [17]. The STROBE guidelines were followed in the reporting of this study [18].

Mostly new data were analyzed. However, of the six coping items of the CAS-1 used in the present study, four and five, were also used in panel network studies of posttraumatic stress disorder (PTSD) symptoms [19] and depressive symptoms [20], respectively. The shortened six-item version of the Difficulties in Emotion Regulation Scale (DERS [21]) used here was also used in the PTSD-study.

The study was carried out in accordance with the provisions of the World Medical Association Declaration of Helsinki and was ethically approved by The Regional Committee for Medical and Health Research Ethics (REK) South East Norway (reference: 125510) and The Norwegian Centre for Research Data (NSD) (reference: 802810). Digital informed consent was obtained from all participants before completing the questionnaire.

### 2.1. Study Design

For the present study, data from the fifth to the eight assessment waves of the MAP-19 survey were specifically chosen because their temporal distance satisfies the statistical assumption of equidistant measures in panel network models [15]. The assessment periods lasted from May 8 to May 25, 2021 (T1), from July 4 to August 1, 2021 (T2), from October 24 to November 12, 2021 (T3), and from January 2 to January 14, 2022 (T4).

### 2.2. Participants and Procedure

The targeted population was adults (age ≥18 years) living in Norway across the period of assessment. Most of the sample (70%) was obtained using a Facebook Business algorithm, and imputed parameters reached a population of 3.6 million adults (target population consisted of 4.2 million Norwegian adults), proportionally targeting each geographic region according to its relative size. To maximize the probability of reaching the residual 600,000 of the population, participants were also recruited through a systematic dissemination of the survey via national, regional, and local information platforms (i.e., television, radio, and newspapers). The exact same online survey was administered to all participants. Participation granted a chance to win a pair of headphones (Bose QuietComfort 35 II). Survey administration errors were assessed by examining the data quality through attention checks and 97.8% of the participants passed the attention check. To ensure high data quality, we excluded subjects failing the attention check. This procedure is explained in detail elsewhere [22]. After this check, a total of 10,061 adults enrolled in the study at its onset (March 2020; 1^st^ data collection wave). The same participants were recontacted and asked to participate at each following assessment.

A stratified representative sample of adults were extracted from the total sample to match the sample as closely as possible to known population demographic characteristics (e.g., equal distribution of sex, education). Within the representative subsample of 4361 participants, 1811 responded at the fifth wave (T1) and only those responding to this wave were included in the analyses. At T2, 35.1% (*n* = 635) of these participants had missing data, decreasing slightly to 32.2% (*n* = 584) at T3. At T4, missingness increased to 47.0% (*n* = 851). To examine the patterns in missingness, logistic regression models were conducted predicting dropout at T2, T3, and T4 based on age, gender, education, and levels of generalized anxiety symptoms at T1. At T2, older participants were less likely to drop out (*β* = −0.012, *p* < 0.001), and males were less likely to drop out than females (*β* = −0.277, *p*=0.009). At T3, higher generalized anxiety symptoms at T5 showed an association with drop out (*β* = 0.026, *p*=0.015). At T8, none of the predictors significantly explained missingness (*p* > 0.10).

Regarding statistical power, the present sample size has shown to be sufficient in simulation studies [15]. Moreover, other panel-GVAR studies using this data had comparable sample sizes and were able to obtain stable model estimates [19, 20].

### 2.3. Measures

The repeatedly measured items are presented in Supporting Information 1. The widely used GAD-7; [23] was selected as a measure of anxiety because it contains individual GAD symptoms, which were the focus of the present study. The first three items capture the two core criteria (A and B) of the DSM-5 GAD [1], the next three reflect three of the six C criteria, and the seventh item is taken from an anxiety scale. Respondents are asked to rate each symptom based on the previous 2 weeks. The items are scored on a four-point Likert scale ranging from 0 (*not at all*) to 3 (*almost every day*). The Norwegian translation used has shown high internal consistency, excellent convergent validity, and indications of discriminant validity [24]. In the present sample, mean Cronbach's alpha across the four assessments was 0.91. Emotion dysregulation was measured with a sum score of six selected items, one from each subscale of the DERS [21]. The CAS-1 [6] was used. This is a 16-item instrument measuring CAS activation during the past week. We included six of the questionnaire's eight maladaptive coping strategies to deal with negative feelings or thoughts as items in the network. CAS-1 Item 1 worry/rumination was not included because worry is already included as a symptom with GAD-7 Item 3. Negative metacognitions entailing danger (Items 9, 11, and 15) was included as a sum score, while Item 13 measuring negative metacognitions entailing uncontrollability (“I cannot control my thoughts”) was excluded from the analysis since GAD-7 Item 2 already measures a similar construct. Positive metacognitions entailing repetitive thinking (Items 10 and 16) were included as a sum score, while positive metacognitions entailing control (Item 14) and attention (Item 12) were included as separate items. Threat monitoring is rated on a 0–8 scale in terms of the amount of time used; the other coping activities are rated on a 0–8 scale in terms of frequency. The strength of belief in the negative and positive metacognitive beliefs are rated on a 0–100 scale.

Three items (Items 15, 7, and 8) of the intolerance of uncertainty scale [25] were included as a sum score in the network. Participants are asked to rate the extent to which each item is characteristic of them on a scale from 1 to 5.

In the present sample, mean Cronbach's alphas across the four assessments were 0.78 for the sum of the six DERS items, 0.70 for negative metacognitive beliefs about danger, 0.51 for positive metacognitive beliefs about repetitive thinking, and 0.83 for intolerance of uncertainty. In addition to questions about sociodemographic characteristics, participants were asked whether they currently received treatment for psychic complaints and whether this treatment was mainly for anxiety, depressive, or stress and trauma-related symptoms.

### 2.4. Statistical Analyses

The panel GVAR model [15] was used to estimate networks from the panel data. Such GVAR models separate (a) a temporal network, representing the average within-person predictive effect between variables from one time period to the next, controlling for all other variables in the model, (b) a contemporaneous network representing the average within-person associations between the variables within the same time period (controlling for all other variables and the temporal effects), and (c) a between-person network representing the associations between the person-means on the variables, controlling for the person-means on the other variables [26]. Panel GVAR models warrant stationary data (i.e., constant means and variances across time). Accordingly, the data were standardized across waves to facilitate stationarity. Because of a close to 4-month time interval between T2 and T3, a dummy (i.e., empty) wave was inserted between these waves to harmonize the data toward a 2-month time-lag across the waves. This is a common strategy adopted in the literature to adhere to the assumption of equidistant measurements (e.g., [27]), allowing a consistent estimation of the 2-month time lag without the need to add or impute any data and maximize the use of already collected data (i.e., through maximum likelihood estimation).

Panel GVAR models use full information maximum likelihood estimation (FIML), which is the state of the art form in cases of missing data, minimizing bias and increasing statistical power by enabling the use of the full range of available data compared to complete case analysis [28]. The method assumes that data are missing at random (MAR). Extensive analyses of this dataset in previous studies, including comparisons between completers and noncompleters at each wave, suggested no systematic or problematic patterns of missingness regarding the MAR assumption [16]. In the specific subset of data used in the present study, Little's missing completely at random (MCAR) test; chi-square = 744.01, *df* = 789, *p*=0.872, failed to reject the hypothesis that data is MCAR.

Recent simulation work has found structural equation modeling (SEM) guidelines for evaluating model fit to be generalizable to network models [29]. SEM guidelines (i.e., comparative fit index [CFI] > 0.90, Tucker Lewis index [TLI] > 0.90, and root mean square error of approximation [RMSEA] < 0.05 [30]) were therefore used. The *psychonetrics* package was used [31] to make the model robust against false positives, we used pruning procedures (at *α* = 0.01) and additional stepup model search along model estimation and the network structures were visualized using the *qgraph* package modification indices [32]. This means that first a saturated model with all edges was estimated, then all nonsignificant edges were removed, and all remaining edges were reestimated. Finally, edges with the largest modification indices were added stepwise until the Bayesian information criterion (BIC) could no longer be improved.

Dynamic network models based on panel data have computational boundaries with respect to power and model estimation and may include up to 30 variables for four waves of data. To keep the number of variables within this limit, sum scores of items were used for negative metacognitions about danger, positive metacognitions about repetitive thinking, emotion dysregulation, and intolerance of uncertainty.

Centrality indices were derived to further describe the dynamics of the network structure [33]. In the temporal network, out-strength centrality is the sum of all outgoing absolute edge weights from a node, whereas in-strength centrality is the sum of all incoming absolute edge weights to a node. In the contemporaneous network, strength centrality denotes the sum of all absolute edge weights connected to a node, highlighting a nodes overall strength of connectivity in the network. Radar plots were used to visualize the different centrality metrics for the dynamic networks, as proposed by [34]. All analyses were conducted in R [35].

## 3. Results

### 3.1. Sample Characteristics

The age of the 1811 participants at the baseline of this study ranged from 18 to 87 years (mean [*M*] = 38.91, standard deviation [*SD*] = 15.18), with the gender distribution of the study being balanced (47.4% female, 52.6% male). Of cultural background, 1680 (92.8%) reported being born in Norway, 94 (5.2%) European or North American, and 37 (2.0%) came from Non-European and North American countries. Hundred (5.5%) of the 1811 participants were first- or second-generation immigrants and belonged to an ethnic minority. The proportion of subjects in each education category (10.6% primary school, 40.8% upper secondary high school, 11.6% student, 37.0% any university degree) largely resembled the Norwegian adult population (e.g., 37.0% in the sample vs. 35.6% in the population having a university degree). However, a smaller proportion of those with compulsory education was represented (10.6% in the sample vs. 24.2% in the population). Furthermore, 1441 (79.6%) reported being currently employed, and 1098 (60.6%) reported being married, in a civil partnership, or in a relationship. Regarding treatment during the pandemic, 93 (5.1%) reported having received it mainly for anxiety, 153 (8.4%) mainly for depressive, and 105 (5.8%) mainly for stress and trauma-related symptoms. The prevalence of participants with current psychiatric disorders at the first assessment in this study was 19.6%, representative of the rate of psychiatric disorders in the Norwegian adult population, which ranges between 16.7% and 25.0% [36]. The sample was furthermore geographically representative of Norway, with the number of participants sampled from each region being proportional to region size.

### 3.2. Networks

**Figure 1:**
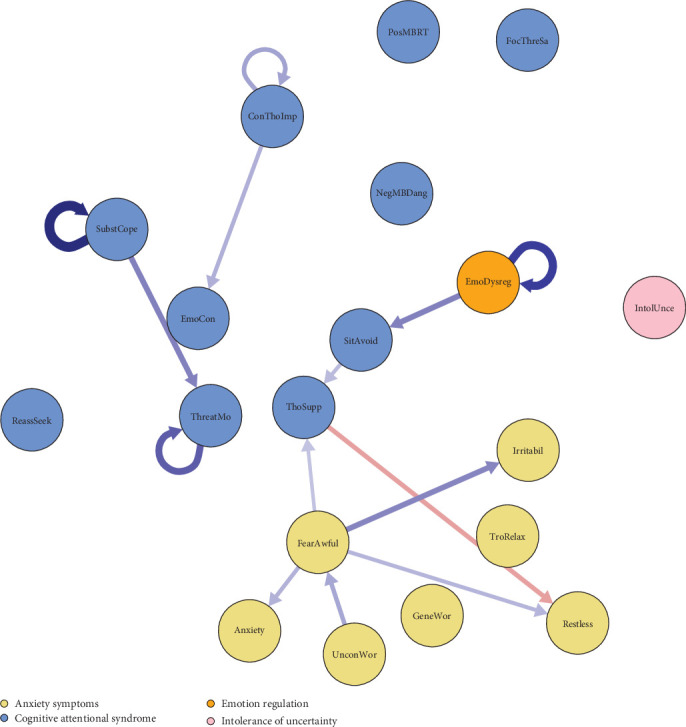
Pruned temporal network of directed associations across time. The temporal network shows the significant (*p*  < 0.001) connections between the nodes, while controlling for all other nodes in the network. The color and thickness of the edges represent their direction (blue = positive, red = negative) and strength, respectively. Nodes are colored according to the domain they belong to.

Mean, SD, and range for the repeatedly measured variables across the four time points are reported in Table S1 in Supporting Information 2. The model fit statistics are reported in Table S2 in Supporting Information 3 and indicate that fit was excellent. The temporal network is presented in Figure 1, and the exact edge weight matrix is presented in Table S3 in Supporting Information 4.

Uncontrollable worry positively predicted future fear of awful events, which in turn predicted increases in anxiety, restlessness, and irritability and the CAS variable thought suppression. Among these predicted variables, thought suppression negatively predicted restlessness. The CAS variable use of substance to cope positively predicted the CAS variable threat monitoring. The variables derived from the other two theoretical perspectives, emotional dysregulation and intolerance of uncertainty, did not predict any GAD symptoms. However, emotional dysregulation positively predicted situational avoidance. Metacognitive beliefs did not predict any other variables over time, except that the belief that it is important to control thoughts positively predicted the CAS variable emotion control.

Autoregressive effects were present for the CAS variables use of substance to cope and threat monitoring, for the meta-belief that controlling thoughts is important, and for emotion dysregulation.

**Figure 2:**
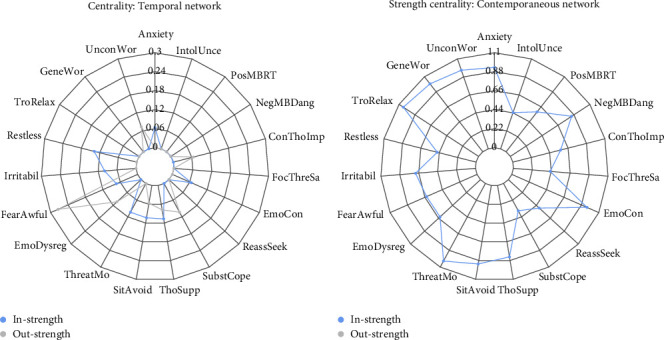
Radar chart showing centrality of the nodes in the temporal (a) and contemporaneous (b) network. Out-strength centrality is the sum of all outgoing absolute edge weights from a node. In-strength centrality is the sum of all incoming absolute edge weights to a node. Strength centrality is the sum of all absolute edge weights connected to a node.

The radar plot in Figure 2(a) shows the in-strength and out-strength estimates for the temporal network. Fear of awful events revealed high out-strength. The GAD symptoms anxiety, restlessness, and irritability, and the CAS variables threat monitoring and situational avoidance had notable in-strength.

**Figure 3:**
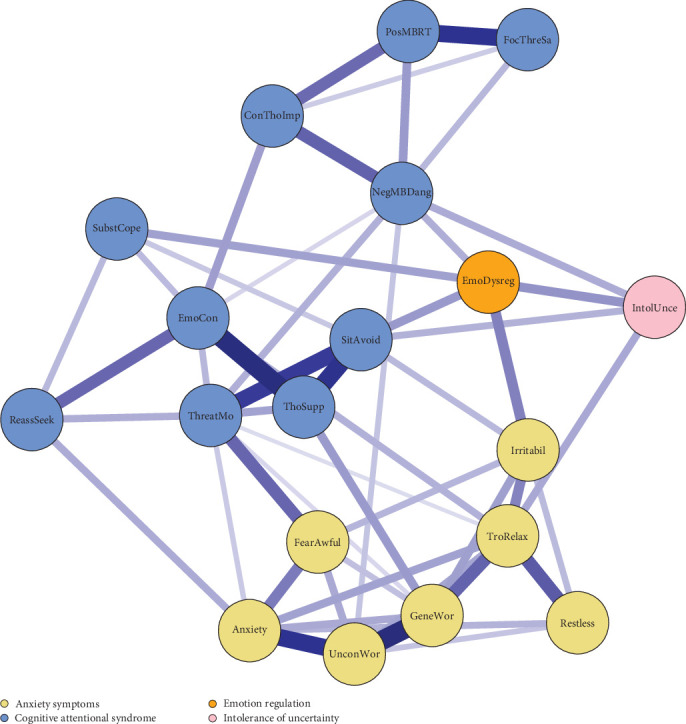
Pruned contemporaneous network of associations within the same time window. The contemporaneous network shows the significant (*p*  < 0.001) connections between nodes, while controlling for all other nodes in the network in addition to controlling for the temporal effects. All edges were positive. The thickness of the edges represent their strength. Nodes are colored according to the domain they belong to.

The contemporaneous network in Figure 3 shows the within-person associations between variables within the same time window. The exact edge weights are included in Table S4 in Supporting Information 4. In contrast to the sparse temporal connections, the contemporaneous network was dense with nodes clustering according to the constructs they belonged to. Notable across construct edges were identified between the symptom fear of awful events and the CAS variable threat monitoring, between the GAD symptom irritability and emotional dysregulation, between the GAD symptom generalized worry and the CAS variable thought suppression, and between the GAD symptom trouble relaxing and intolerance of uncertainty.

The radar plot in Figure 2 shows that many variables had high strength centrality. As preregistered, the present study is focused on within-person relationships. Accordingly, the between-person network is presented in Figure S1 in Supporting Information 5 to provide full information of all models.

## 4. Discussion

The main purpose of this study was to investigate the long-term dynamic interactions between symptoms of GAD and their theorized mechanistic processes derived from three treatment models of GAD: the emotion dysregulation model [5], the model underlying MCT [6], and the intolerance of uncertainty model [7]. The 2-month intervals for assessment seem to be a reasonable starting point for investigating these more long-term relationships as this time scale seems appropriate for the known courses and fluctuations of GAD and GAD symptoms [1].

Consistent with some of the processes stipulated in the metacognitive model [6], the experience (and probable associated belief) that worry is uncontrollable predicted the GAD symptom fear of awful events, which in turn predicted a range of other GAD symptoms, that is, anxiety, restlessness, and irritability. These findings may reflect that out of control worry involves a runaway process that ends in fears that events with strong negative valence are highly probable. On the other hand, the hypothesis that the experience of uncontrollability of worry is taken as a sign of loss of mind was not supported. In line with the MCT model, more use of substance to regulate negative feelings and thoughts predicted more threat monitoring at the next timepoint. Inconsistent with the MCT model, the coping strategy thought suppression did not positively predict any symptoms but, on the contrary, was predicted by the symptom fear of awful events. Also inconsistent with the MCT model, thought suppression *negatively* predicted restlessness.

The contemporaneous network represents within-persons relationships between symptoms and theory-derived variables within a 2-months period. They may represent relationships that unfold on a shorter or longer timescale than 2 months. The results were consistent with the proposals of the metacognitive model that more threat monitoring is related to more fear of awful events and more thought suppression to more generalized worry. That is, more threat monitoring is associated with detection of more signs that something awful is going to happen, and more thought suppression is tied to increases in intrusive and worrisome thoughts. However, it should be noted that the contemporaneous results do not inform about the direction of the obtained relationships.

The temporal findings suggest that emotion dysregulation may be an important mechanism for avoidance. The contemporaneous relationship between dysregulation and irritability is consistent with findings on an inter-individual level that difficulties experiencing, tolerating, and processing emotions are associated with irritability [37].

No relationships proposed by the intolerance of uncertainty model of GAD were identified in the temporal network. Thus, the finding of several predictive relationships of intolerance of uncertainty on a daily time level [14] was not replicated over the 2-month time-lag of the present study. However, greater intolerance of uncertainty was related to more trouble relaxing in the contemporaneous network, suggesting that intolerance may specifically be related to this symptom of GAD.

Regarding the interaction within the GAD symptoms cluster, the findings supported that uncontrollability of worry, fear of awful events, anxiety, restlessness, and irritability are components of GAD in the sense that they were parts of a dynamic network of positive predictive relationships. The DSM defined core symptom generalized worry and trouble relaxing were unrelated to other symptoms. It is noteworthy that fear of awful events, which is derived from anxiety scales, was more central than generalized worry in this study. Thus, in a within-individual diagnostics supplementary to the inter-individual DSM, the findings suggest that uncontrollability of worry and fear of awful events should be taken as central symptoms of GAD.

The limitations of this study include that the variables were assessed by self-report and that a fewer proportion of those with only primary school were represented in the sample. Because the study was conducted during the pandemic, it is unclear whether the findings can be generalized to nonpandemic settings. However, the study focused on the dynamics of symptoms and mechanisms and these internal dynamics may unfold across various situations involving different types of stressors (e.g., pandemic, stressful life events). The three models were tested in different ways as multiple item scores were used to examine the MCT model and single sum scores were used to examine emotion dysregulation and intolerance of uncertainty. Such higher-level constructs are broad and may encompass various lower-level processes, which may have been disguised in the present networks. On the other hand, use of sum scores may involve more power to detect broader relationships between several process variables and specific symptoms. The standardization of data across waves may have led to inflated fit measures. Strengths of this study include the use of a state of the art panel approach allowing the estimation of temporal networks on a larger time scale and the large sample size which contributed to the accuracy and stability of the network estimates. In contrast to traditional latent variable approaches, the network approach focuses on observable symptoms and related observable processes. It further embodies an interactive approach, focusing on how symptoms and psychological mechanisms intertwine and engage in vicious cycle to maintain psychopathology. Theoretically and across different types of applications, this approach also provides more specific hypothesis about actionable targets that can provide clinical guidance and personalized treatment [38]. It also forms a more granular basis for the understanding of psychopathology [39].

## 5. Conclusion

The findings indicate the importance of the theory-derived variables, the experience and belief that worry is uncontrollable and emotion dysregulation as potential targets for intervention to alleviate GAD symptoms. The findings also indicate that uncontrollability of worry and fear of awful events should be considered central symptoms of GAD in a within-individual diagnostics supplementary to DSM. How more specific dimensions of emotion dysregulation and intolerance of uncertainty relate to GAD symptoms would advance the literature and should be pursued in future research. The present study assessed symptoms and processes on a single time scale. Future studies should connect frequent measurements of rapidly cycling emotions with less frequent measurements of moods and negative cognitions about the self and future [40]. As the pandemic context may have moderated the internal dynamics of symptoms and mechanisms, the study should be replicated in nonpandemic settings. More in general, the present network approach might be integrated with transdiagnostic dimensional approaches and clinical stage approaches [41]. For instance, network theories of GAD could be refined by incorporating notions about common causes that are a major focus of transdiagnostic dimensional approaches. Moreover, network approaches might help explain the dynamics of different stages of GAD development.

## Data Availability

Access to the data can be granted from the principal investigators Omid V. Ebrahimi and Sverre Urnes Johnson following ethical approval of a suggested project plan for the use of data granted by NSD and Regional Committee for Medical and Health Research Ethics (REK).

## Supporting Information

Supporting Information 1 List of repeatedly measured items.

Supporting Information 2 Descriptives of the repeatedly measured items. Table S1: Descriptive statistics on the network variables.

Supporting Information 3 Model fit statistics. Table S2: Model fit statistics for the saturated and pruned model.

Supporting Information 4 Edge weights for the temporal and contemporaneous network. Table S3: Edge weights between nodes in the temporal network. Table S4: Edge weights between nodes in the contemporaneous network.

Supporting Information 5 Between-person network. Figure S1: Between-person network of associations between the person-means on the variables.
