# Supplementary material for: Mapping the Dynamics of Generalized Anxiety Symptoms and Actionable Transdiagnostic Mechanisms: A Panel Study
Source: Depress Anxiety. 2025 May 13;2025:1885004. doi: 10.1155/da/1885004 (PMC12092150; doi:10.1155/da/1885004)
Supplement: Supporting Information 1 — List of repeatedly measured items. [file 1885004.f1.docx]

# **Supplementary 1**

Hoffart, A., Skjerdingstad, N., Freichel, R., Johnson, S. U., Epskamp, S., &

Ebrahimi, O. V. Mapping the Dynamics of Generalized Anxiety Symptoms and Actionable Transdiagnostic Mechanisms – A Panel Study

**List of repeatedly measured items**

*Emotion dysregulation* (EmoDysreg; sum of 6 items from the Difficulties in Emotion Regulation Scale (DERS; Gratz and Roemer, 2004)):

“When I am upset, I become irritated, feel ashamed or feel guilty for feeling that way”, (from the Non-acceptance of emotional responses subscale, item 21, 25, 29 combined).

“When I am upset, I have difficulties getting work done”, (from the Difficulty engaging in goal-directed behavior subscale, item 13).

“When I am upset, I lose control over my behaviors”, (from the Impulse control difficulties subscale, item 32).

“When I am upset, I acknowledge my emotions”, (from the Lack of emotional awareness subscale, item 10, reversed).

“When I am upset, I believe there is nothing I can do to make myself feel better”, (from the Limited access to emotion regulation strategies subscale, item 28).

“I have difficulty making sense of my feelings”, (from the Lack of emotional clarity subscale, item 5).

From the Cognitive-Attentional Syndrome – 1 (CAS-1; Wells, 2009):

*Threat monitoring* (ThreatMon):

“How much time in the last week have you been focusing attention on the things you find threatening (e.g., symptoms, thoughts, danger)?” (item 2)

“How often in the last week have you done the following in order to cope with your negative feelings or thoughts?:

*Situational avoidance* (SitAvoid: “Avoided situations”) (item 3)

*Thought suppression* (ThoSupp: “Tried not to think about things”) (item 4)

*Use of substance to cope* (SubstCope: “Used alcohol/drugs”) (item 5)

*Reassurance seeking* (ReassSeek: “Asked for reassurance”) (item 6)

*Emotion control* (EmoCon: “Tried to control my emotions”) (item 7)

*Negative metabeliefs about danger* (NegMBDang: items 9, 11, 15):

“Worrying too much could harm me” (item 9)

“Strong emotions are dangerous” (item 11)

“Some thoughts could make me lose my mind (item 15)

*Positive metabeliefs about repetitive thinking* (PosMBRT: items 10, 16):

“Worrying helps me cope” (item 10)

“Analyzing my problems will help me find answers” (item 16)

*Focus on threat makes safe* (FocThreSa: item 12):

“Focusing on possible threat can keep me safe”

*Control thoughts important* (ConThoImp: item 14):

“It is important to control my thoughts”

*Intolerance of uncertainty* (IntolUnce; sum of 3 items from the Intolerance of Uncertainty Scale (IUS; Buhr and Dugas, 2002)):

“When I am uncertain, I cannot function very well” (item 15)

“Unforeseen events upset me greatly” (item 7)

“It frustrates me not having all the information I need” (item 8)

**References**

Buhr, K., Dugas, M.J., 2002. The Intolerance of Uncertainty Scale: Psychometric properties of the English version. Behav. Res. Ther. 40(8), 931-946. <https://doi.org/10.1016/S0005-7967(01)00092-4>

Gratz, K.L., Roemer, L. (2004). Multidimensional Assessment of Emotion Regulation and Dysregulation: Development, Factor Structure, and Initial Validation of the Difficulties in Emotion Regulation Scale. J. Psychopathol. Behav. Assess. 26, 41–54. <https://doi.org/10.1023/B:JOBA.0000007455.08539.94>

Wells, A. (2009). Metacognitive therapy for anxiety and depression. Guilford Press, New York.
