# Supplementary material for: Mapping the Dynamics of Generalized Anxiety Symptoms and Actionable Transdiagnostic Mechanisms: A Panel Study
Source: Depress Anxiety. 2025 May 13;2025:1885004. doi: 10.1155/da/1885004 (PMC12092150; doi:10.1155/da/1885004)
Supplement: Supporting Information 2 — Descriptives of the repeatedly measured items. Table S1: Descriptive statistics on the network variables. [file 1885004.f2.docx]

# **Supplementary 2**

Hoffart, A., Skjerdingstad, N., Freichel, R., Johnson, S. U., Epskamp, S., &

Ebrahimi, O. V. Mapping the Dynamics of Generalized Anxiety Symptoms and Actionable Transdiagnostic Mechanisms – A Panel Study

| **Descriptives of the repeatedly measured items**  **Table S1**  *Descriptive Statistics on the Network Variables* | | | |  |
| --- | --- | --- | --- | --- |
| Variable | Timepoint | *M* | *SD* | Range |
| **Anxiety** | T1 | .76 | .83 | 0–3 |
| *1 item* | T2 | .60 | .76 | 0–3 |
|  | T3 | .61 | .77 | 0–3 |
|  | T4 | .66 | .81 | 0–3 |
| **Uncontrollable worry** | T1 | .64 | .84 | 0–3 |
| *1 item* | T2 | .49 | .72 | 0–3 |
|  | T3 | .50 | .76 | 0–3 |
|  | T4 | .57 | .81 | 0–3 |
| **Generalized worry** | T1 | .91 | .88 | 0–3 |
| *1 item* | T2 | .73 | .79 | 0–3 |
|  | T3 | .74 | .82 | 0–3 |
|  | T4 | .81 | .85 | 0–3 |
| **Trouble relaxing** | T1 | .92 | .92 | 0–3 |
| *1 item* | T2 | .77 | .87 | 0–3 |
|  | T3 | .78 | .87 | 0–3 |
|  | T4 | .82 | .90 | 0–3 |
| **Restlessness** | T1 | .49 | .73 | 0–3 |
| *1 item* | T2 | .39 | .67 | 0–3 |
|  | T3 | .36 | .66 | 0–3 |
|  | T4 | .39 | .67 | 0–3 |
| **Irritability** | T1 | .94 | .80 | 0–3 |
| *1 item* | T2 | .77 | .75 | 0–3 |
|  | T3 | .76 | .74 | 0–3 |
|  | T4 | .82 | .77 | 0–3 |
| **Fear awful events** | T1 | .45 | .71 | 0–3 |
| *1 item* | T2 | .38 | .69 | 0–3 |
|  | T3 | .36 | .67 | 0–3 |
|  | T4 | .39 | .69 | 0–3 |
| **Emotion dysregulation** | T1 | 12.29 | 4.35 | 6–28 |
| *6 items* | T2 | 11.71 | 4.19 | 6–30 |
|  | T3 | 11.70 | 4.24 | 6–28 |
|  | T4 | 11.82 | 4.37 | 6–30 |
| **Threat monitoring** | T1 | 1.78 | 1.92 | 0–8 |
| *1 item* | T2 | 1.56 | 1.80 | 0–8 |
|  | T3 | 1.84 | 1.89 | 0–8 |
|  | T4 | 1.78 | 1.97 | 0–8 |
| **Situational avoidance** | T1 | 1.77 | 2.04 | 0–8 |
| *1 item* | T2 | 1.48 | 1.84 | 0–8 |
|  | T3 | 1.60 | 1.88 | 0–8 |
|  | T4 | 1.64 | 2.00 | 0–8 |
| **Thought suppression** | T1 | 2.34 | 2.20 | 0–8 |
| *1 item* | T2 | 1.94 | 2.06 | 0–8 |
|  | T3 | 2.22 | 2.14 | 0–8 |
|  | T4 | 2.17 | 2.23 | 0–8 |
| **Substance to cope** | T1 | .59 | 1.38 | 0–8 |
| *1 item* | T2 | .54 | 1.39 | 0–8 |
|  | T3 | .47 | 1.19 | 0–8 |
|  | T4 | .55 | 1.39 | 0–8 |
| **Reassurance seeking** | T1 | .96 | 1.63 | 0–8 |
| *1 item* | T2 | .87 | 1.51 | 0–8 |
|  | T3 | .98 | 1.59 | 0–8 |
|  | T4 | .99 | 1.66 | 0–8 |
| **Emotion control** | T1 | 2.14 | 2.20 | 0–8 |
| *1 item* | T2 | 1.80 | 2.08 | 0–8 |
|  | T3 | 1.95 | 2.08 | 0–8 |
|  | T4 | 1.93 | 2.19 | 0–8 |
| **Negative metabeliefs** | T1 | 84.86 | 69.10 | 0–300 |
| *3 items* | T2 | 75.75 | 68.67 | 0–300 |
|  | T3 | 79.28 | 68.63 | 0–300 |
|  | T4 | 78.57 | 69.61 | 0–300 |
| **Positive metabeliefs** | T1 | 80.76 | 43.92 | 0–200 |
| *2 items* | T2 | 82.70 | 45.47 | 0–200 |
|  | T3 | 85.78 | 44.45 | 0–200 |
|  | T4 | 84.65 | 45.32 | 0–200 |
| **Focus on threat** | T1 | 21.33 | 24.21 | 0–100 |
| *1 item* | T2 | 22.30 | 24.77 | 0–100 |
|  | T3 | 24.81 | 25.63 | 0–100 |
|  | T4 | 26.12 | 24.92 | 0–100 |
| **Control thoughts important** | T1 | 40.56 | 30.86 | 0–100 |
| *1 item* | T2 | 37.53 | 30.00 | 0–100 |
|  | T3 | 38.44 | 30.22 | 0–100 |
|  | T4 | 37.64 | 29.89 | 0–100 |
| **Intolerance of Uncertainty** | T1 | 7.55 | 3.16 | 3–15 |
| *3 items* | T2 | 7.47 | 3.14 | 3–15 |
|  | T3 | 7.52 | 3.09 | 3–15 |
|  | T4 | 7.57 | 3.21 | 3–15 |
