# Supplementary material for: Mapping the Dynamics of Generalized Anxiety Symptoms and Actionable Transdiagnostic Mechanisms: A Panel Study
Source: Depress Anxiety. 2025 May 13;2025:1885004. doi: 10.1155/da/1885004 (PMC12092150; doi:10.1155/da/1885004)
Supplement: Supporting Information 3 — Model fit statistics. Table S2: Model fit statistics for the saturated and pruned model. [file 1885004.f3.docx]

# **Supplementary 3**

Hoffart, A., Skjerdingstad, N., Freichel, R., Johnson, S. U., Epskamp, S., &

Ebrahimi, O. V. Mapping the Dynamics of Generalized Anxiety Symptoms and Actionable Transdiagnostic Mechanisms – A Panel Study

**Model fit statistics**

**Table S2**

*Model Fit Statistics for the Saturated and Pruned Model*

|  | *X^2^* | RMSEA | TLI/CFI | AIC | BIC |
| --- | --- | --- | --- | --- | --- |
| Saturated Model | 3320.60 | .014 | .97/.98 | 200413.74 | 204594.99 |
| Prune + stepup | 5440.57 | .021 | .94/.94 | 201349.89 | 202224.65 |
