# Supplementary material for: Mapping the Dynamics of Generalized Anxiety Symptoms and Actionable Transdiagnostic Mechanisms: A Panel Study
Source: Depress Anxiety. 2025 May 13;2025:1885004. doi: 10.1155/da/1885004 (PMC12092150; doi:10.1155/da/1885004)
Supplement: Supporting Information 4 — Edge weights for the temporal and contemporaneous network. Table S3: Edge weights between nodes in the temporal network. Table S4: Edge weights between nodes in the contemporaneous network. [file 1885004.f4.docx]

# **Supplementary 4**

Hoffart, A., Skjerdingstad, N., Freichel, R., Johnson, S. U., Epskamp, S., &

Ebrahimi, O. V. Mapping the Dynamics of Generalized Anxiety Symptoms and Actionable Transdiagnostic Mechanisms – A Panel Study

**Edge weights for the temporal and contemporaneous network**

**Table S3**

*Edge Weights between Nodes in the Temporal Network*

**

*Note.* A node’s n horizontal line presents its temporal prediction of the other nodes. A node’s vertical line presents the other nodes’ temporal prediction of the node. The diagonal presents the nodes’ autoregressive effects. Nodes: 1; Anxiety; 2; UnconWor; 3; GeneWor, 4; TroRelax, 5; Restless, 6; Irritabil, 7; FearAwful, 8; EmoDysreg, 9; ThreatMo, 10; SitAvoid, 11; ThoSupp, 12; SubstCope, 13; ReassSeek, 14; EmoCon, 15; FocThreSa, 16; ConThoImp, 17; NegMBDang, 18; PosMBRT, 19; IntolUnce.

**Table S4**

*Edge Weights between Nodes in the Contemporaneous Network*

*Note.* Nodes: 1; Anxiety; 2; UnconWor; 3; GeneWor, 4; TroRelax, 5; Restless, 6; Irritabil, 7; FearAwful, 8; EmoDysreg, 9; ThreatMo, 10; SitAvoid, 11; ThoSupp, 12; SubstCope, 13; ReassSeek, 14; EmoCon, 15; FocThreSa, 16; ConThoImp, 17; NegMBDang, 18; PosMBRT, 19; IntolUnce.
