# Supplementary material for: Mapping the Dynamics of Generalized Anxiety Symptoms and Actionable Transdiagnostic Mechanisms: A Panel Study
Source: Depress Anxiety. 2025 May 13;2025:1885004. doi: 10.1155/da/1885004 (PMC12092150; doi:10.1155/da/1885004)
Supplement: Supporting Information 5 — Between-person network. Figure S1: Between-person network of associations between the person-means on the variables. [file 1885004.f5.docx]

# **Supplementary 5**

Hoffart, A., Skjerdingstad, N., Freichel, R., Johnson, S. U., Epskamp, S., &

Ebrahimi, O. V. Mapping the Dynamics of Generalized Anxiety Symptoms and Actionable Transdiagnostic Mechanisms – A Panel Study

**Between-person Network**

**Fig. S1**

*Between-Person Network of Associations between the Person-Means on the Variables*

*Note.* The edges of the between-person network represent the correlations between the person-means on the variables, given the person-means on the other variables. Variables (nodes): Anxiety = anxiety, UnconWor = uncontrollability of worry, GeneWor = generalized worry, TroRelax = trouble relaxing, Restless = restlessness, Irritabil = irritability, FearAwful = fear awful events, EmodDysreg = emotion dysregulation, ThreatMon = threat monitoring, SitAvoid = situational avoidance, ThoSupp = thought suppression, SubstCope = substance to cope, ReassSeek = reassurance seeking, EmoCon = emotion control, NegMBDang = negative metabeliefs about danger, PosMBRT = positive metabeliefs about repetitive thinking, FocThreSa = focus on threat makes safe, ConThoImp = control thoughts important, IntolUnce = intolerance of uncertainty.
